# Systemic lupus erythematosus

**DOI:** 10.1186/1750-1172-1-6

**Published:** 2006-03-27

**Authors:** Jessica J Manson, Anisur Rahman

**Affiliations:** 1 Centre for Rheumatology Research, Windeyer Building, University College London,46 Cleveland Street, London W1T 4JF, UK

## Abstract

Systemic lupus erythematosus (SLE) is a clinically heterogeneous disease, which is autoimmune in origin and is characterized by the presence of autoantibodies directed against nuclear antigens. It is a multi-system disease, and patients can present in vastly different ways. Prevalence varies with ethnicity, but is estimated to be about 1 per 1000 overall with a female to male ratio of 10:1. The clinical heterogeneity of this disease mirrors its complex aetiopathogenesis, which highlights the importance of genetic factors and individual susceptibility to environmental factors. SLE can affect every organ in the body. The most common manifestations include rash, arthritis and fatigue. At the more severe end of the spectrum, SLE can cause nephritis, neurological problems, anaemia and thrombocytopaenia. Over 90% of patients with SLE have positive anti-nuclear antibodies (ANA). Significant titres are accepted to be of 1:80 or greater. SLE is a relapsing and remitting disease, and treatment aims are threefold: managing acute periods of potentially life-threatening ill health, minimizing the risk of flares during periods of relative stability, and controlling the less life-threatening, but often incapacitating day to day symptoms. Hydroxychloroquine and non-steroidal anti-inflammatory drugs are used for milder disease; corticosteroids and immunosuppressive therapies are generally reserved for major organ involvement; anti-CD20 monoclonal antibody is now used in patients with severe disease who has not responded to conventional treatments. Despite enormous improvements in prognosis since the introduction of corticosteroids and immunosuppressive drugs, SLE continues to have a significant impact on the mortality and morbidity of those affected.

## Disease name and synonyms

Systemic lupus erythematosus

Lupus

## Definition/Diagnostic criteria

Systemic lupus erythematosus (SLE) is a clinically heterogeneous disease which is autoimmune in origin, and characterized by the presence of autoantibodies directed against nuclear antigens. It is, by definition, a multi-system disease, and patients can present in vastly different ways. Classification criteria have been developed, in part in an attempt to keep the patient group as homogeneous as possible for research purposes.

These criteria (Table 1), which are published by the American College of Rheumatology (ACR), were revised in 1982 [1] and combine clinical signs and symptoms with abnormalities detected in blood tests such as a positive anti-nuclear antibody or thrombocytopaenia. They were further updated in 1997 [2] to reflect a greater understanding of the role of antiphospholipid antibodies in patients with SLE.

**Table 1:** Diagnostic criteria of SLE. Adapted from Tan *et al*, 1982 [1]. A person is said to have SLE if he/she meets any 4 of these 11 criteria simultaneously or in succession

**Criterion**	**Definition/examples**
1. Malar rash	Fixed erythema over the malar eminences, tending to spare the nasolabial folds
2. Discoid rash	Erythematosus raised patches, may scar
3. Photosensitivity	Skin rash as a result of unusual reaction to sunlight
4. Oral ulcers	Usually painless
5. Arthritis	Non-erosive: Jaccoud's arthropathy
6. Serositis	a) Pleuritis – pleuritic pain, pleural rub, pleural effusion b) Pericarditis – ECG changes, rub, pericardial effusion
7. Renal disorder	a) Proteinuria (> 3+ or 0.5 g/day) b) Cellular casts in urine
8. Neurological disorder	a) Seizures b) Psychosis
9. Haematological disorder	a) Haemolytic anaemia b) Leukopaenia c) Lymphopaenia d) Thrombocytopaenia
10. Immunological disorder	a) Anti-DNA antibodies b) Anti-Sm antibodies c) Anti-phospholipid antibodies
11. Anti-nuclear antibody	Exclude drug causes

## Epidemiology

SLE is up to 10 times more common in women than men, and typically has a predilection for women in their child-bearing years [3]. Reliable data about the prevalence of SLE are difficult to come by. Variable methods for data collection and inconsistency regarding case definition contribute to this problem, but it is clear that the statistics vary with ethnicity. The overall prevalence is estimated to be about 1 per 1000. A study from Birmingham, UK, found the prevalence to be 27.7/100,000 in the general population, but nearly 9 times higher in Afro-Caribbean females [4]. Data from a national health survey in the USA found the self-reported prevalence of SLE (defined as having been given a diagnosis of SLE by a physician) to be 241/100,000 [5]. Recognizing that this may well be an over-estimate, combining self-reporting with evidence of a current prescription for anti-malarials, corticosteroids, or other immunosuppressive medications reduced this figure to 53.6/100,000 [5].

## Aetiology/Pathogenesis

The clinical heterogeneity of this disease is mirrored by its complex aetiopathogenesis (reviewed in [6]). Twin studies initially indicated the importance of genetic factors, and genome screening has highlighted a number of potential loci of interest [7]. In the susceptible individual, disease may result from a variety of environmental triggers including exposure to sunlight, drugs and infections, particularly with Epstein-Barr virus. Even within one patient, lupus flares can result from different precipitants at different times.

Despite extensive work, the precise pathological mechanisms of SLE are still not fully understood. The majority of patients have elevated levels of autoantibodies, directed in particular against nuclear components such as nucleosomes, DNA and histones, and it is generally accepted that at least some of these have a directly pathogenic role, either by precipitating as immune complexes in target organs or by cross-reacting with other functionally relevant antigens. The presence and persistence of these autoantibodies indicate an abnormality in tolerance, which results from a combination of abnormal handling of autoantigens following apoptosis, and deranged function of T and B lymphocytes.

## Differential diagnosis

The list of possible differential diagnoses is broad, and will vary with the presentation of each case. The non-specific clinical features of widespread pain and fatigue mean that in some cases fibromyalgia and other chronic pain syndromes may be appropriate differentials. Indeed, it is important to note that fibromyalgia and SLE can co-exist in the same patient.

A number of patients will present with a cluster of features suggestive of an autoimmune rheumatic disease, though at initial presentation the final diagnosis appears unclear. A proportion of these "undifferentiated" patients will go on to develop full blown SLE, or other diseases such as systemic sclerosis.

Some malignancies, particularly lymphoma and leukaemia, which are relevant to this age-group, can present with a similar clinical picture. Similarly, there is significant overlap with the presentation of some infections, notably, tuberculosis, HIV/AIDS and bacterial endocarditis. In view of the immunosuppressive nature of the required drugs, it is clearly crucial to exclude underlying infection before starting treatment for SLE.

### The acutely ill patient

Even when the diagnosis of SLE has been established, the acutely ill patient must be thoroughly assessed before the illness is presumed to be due to a flare of their lupus. Since both SLE itself and the drugs used to treat it can cause immunosuppression, sepsis is common and may present in atypical ways. Thus, the physician must remain vigilant in looking for infection. In addition, the possibility of catastrophic antiphospholipid syndrome should be considered. We are becoming increasingly aware of this rare, but devastating association. A recent paper [8] describes a series of 80 such patients. The occlusion of multiple small vessels results in multi-organ failure, and mortality was reported to be 48% in this group.

## Clinical manifestations

The clinical features of SLE are diverse and will be discussed by system as much as possible, and where appropriate, each section will refer to a review for more information. Quoted frequencies of each disease manifestation come from a prospective European study which followed 1000 patients with SLE over 10 years [3].

***Constitutional symptoms ***such as fatigue, weight loss and fever are not life threatening, but have a significant impact on quality of life. Patients with SLE describe overwhelming fatigue and unsatisfying sleep, though the extent to which this tiredness relates directly to lupus disease activity remains controversial [9].

***Renal disease ***affects about 30% of patients with SLE, and remains the most dangerous, life-threatening complication. Patients who will develop lupus nephritis most commonly do so within the first few years of their disease. As renal involvement is often asymptomatic, particularly initially, regular urinalysis and blood pressure monitoring is crucial. Renal involvement is characterized by proteinuria (> 0.5 g/24 hours), and/or red cell casts, and early referral for renal biopsy is generally advocated. The histological classification of lupus nephritis has recently been updated [10]. Table 3 shows the revised classification criteria, developed under the auspices of the International Society of Nephrology and the Renal Pathology Society. Lupus nephritis classes I-V describe mesangial (I, II), proliferative (III, IV) or membranous (V) lesions, and each biopsy may have features of more than one class of disease. Classes III and IV are subdivided further depending on the activity or chronicity of the abnormalities seen. Class VI is reserved for widespread sclerotic disease. The renal biopsy findings are used to assess prognosis and guide management. Response to treatment can be assessed using serial urine protein/creatinine ratios, in addition to other more general measures of disease activity (see below).

**Table 2:** The revised classification of glomerulonephritis in SLE [10]

**Class I**	**Minimal mesangial lupus nephritis **Normal on light microscopy. Mesangial immune deposits on immunofluorescence
**Class II**	**Mesangial proliferative lupus nephritis **Mesangial hypercellularity or matrix expansion, with mesangial immune deposits on immunofluorescence
**Class III**	**Focal lupus nephritis **Glomerulonephritis involving < 50% of glomeruli, typically with sub-endothelial immune deposits.
**Class IV**	**Diffuse lupus nephritis **Glomerulonephritis involving > 50% of glomeruli, typically with sub-endothelial immune deposits. Can be segmental or global.
**Class V**	**Membranous lupus nephritis **Global or segmental sub-epithelial immune deposits
**Class VI**	**Advanced sclerotic lupus nephritis **> 90% of glomeruli globally sclerosed without residual activity

**Table 3:** Neuropsychiatric syndromes seen in systemic lupus erythematosus [11]

**Central nervous system**	**Peripheral nervous system**
Aseptic meningitis	Acute inflammatory polyneuropathy
Cerebrovascular disease	Autonomic disorder
Demyelinating syndrome	Mononeuropathy (single or multiplex)
Headache	Myasthenia gravis
Movement disorder	Cranial neuropathy
Myelopathy	Plexopathy
Seizure disorders	Polyneuropathy
Acute confusional state	
Anxiety disorder	
Cognitive dysfunction	
Mood disorder	
Psychosis	

***Neuropsychiatric lupus ***(NPSLE) is seen in about 20% of cases. NPSLE is often a difficult diagnosis to make. Not only are there 19 different clinical manifestations as described by the American College of Rheumatology [11] (Table 3), but there is also no single, simple diagnostic test. In many cases, a brain biopsy would be the only definitive test, and this is rarely performed. The clinical features vary from central nervous system disease causing headache and seizures, or psychiatric diagnoses including depression and psychosis, to peripheral nervous system involvement causing neuropathy.

The investigations of choice will vary with the presentation. Central nervous system disease usually warrants magnetic resonance imaging (MRI) of brain or spinal cord, and examination of the cerebrospinal fluid where appropriate. It must be remembered, however, that normal investigations, and lack of evidence of disease activity in another system, do not rule out the diagnosis of NPSLE – in a recent study of MRI in patients with NPSLE, 34% had normal brain scans [12]. This included patients with focal disease clinically. Interestingly, only one of the 85 patients included in this study proceeded to brain biopsy, which is probably indicative of generally accepted practice.

The frequency of ***musculoskeletal disease ***in SLE means that rheumatologists often make the initial diagnosis. Arthralgia and myalgia occur in most patients. The classical "Jaccoud's arthropathy" although not causing a destructive arthritis, can result in significant deformity and functional impairment. A rheumatoid-like arthritis is seen more rarely, sometimes associated with a positive rheumatoid factor. Similarly, an overlap with myositis also occurs.

***Skin involvement ***in lupus is also very common. In addition to the classic malar and discoid rashes, more generalized photosensitivity is often present, and furthermore sun exposure is known to trigger systemic disease flares. Alopecia can be scarring when associated with discoid lesions, or more diffuse, often fluctuating with disease activity. Recurrent crops of mouth ulcers are also a feature of active disease. Other oral manifestations include dryness as a result of secondary Sjogren's syndrome, and these patients also experience dryness of the eyes and vagina.

***Haematological features ***include normocytic normochromic anaemia, thrombocytopaenia (sometimes, but not always associated with antiphospholipid antibodies) and leukopaenia. Severe haematological disease can occur, but this is relatively rare [13].

***Pleuritis ***, causing chest pain, cough and breathlessness, is the most common pulmonary manifestation of SLE [14]. Although pleuritic symptoms may relate directly to active lupus, pulmonary embolism must always be considered, particularly in those who have antiphospholipid antibodies. Pleural effusions are usually exudates, have low levels of complement, and test positive for anti-nuclear antibodies (ANA). Infections are common, and any parenchymal lesion must be treated as infectious until proven otherwise. Rarer complications include interstitial lung disease and pulmonary hypertension (both more common in systemic sclerosis) and pulmonary haemorrhage.

***Gastrointestinal involvement ***[15] most commonly results in non-specific abdominal pain and dyspepsia though it can be unclear whether such pain results from the illness itself or from drug side-effects. Hepatosplenomegaly can come and go with disease activity. Mesenteric vasculitis is very rare, but can be life-threatening, especially if it leads to perforation, and may only be diagnosed at laparotomy.

SLE is associated with a variety of ***vascular manifestations***. Raynaud's phenomenon, causing the classical triphasic colour change, was seen in 16% of patients in the European study[3]. Abnormalities in the micro vasculature are also thought to account for the association with livedo reticularis. Arterial and venous thrombosis affected up to 10% of the cohort, particularly in association with the secondary antiphospholipid syndrome.

In the last decade, it has become clear that patients with SLE are at increased risk of ***atherosclerosis***. Chronic inflammation and the use of corticosteroids contribute to this risk, and have led rheumatologists to treat SLE as an independent risk factor for stroke and myocardial infarction, much as an endocrinologist might regard the risk associated with diabetes. Ward [16] showed that in women between 18 and 44 years of age, those with SLE were twice as likely to develop a myocardial infarction or stroke, and nearly 4 times as likely to present with heart failure.

Screening for ***cardiac disease ***with echocardiography (ECHO) has established that asymptomatic valvular lesions are common. In addition, pericarditis and pericardial effusions are common though myocardial disease is relatively rare.

## Laboratory findings

Over 90% of patients with SLE have positive anti-nuclear antibodies (ANA). Significant titres are accepted to be of 1:80 or greater. ANA although sensitive, is far from specific for SLE. A positive ANA is also seen in many other illnesses including systemic sclerosis and polymyositis, as well as some chronic infections. All patients should be screened for extractable nuclear antigens (ENA). Different ENAs are associated with different disease manifestations – for instance, anti-Sm is associated with renal involvement, and anti-Ro with secondary Sjogren's syndrome.

Antibodies to double-stranded DNA (dsDNA), and more recently to nucleosomes (though this test is not commonly available in most routine labs) are more specific for SLE, and anti-dsDNA titres are also predictive of renal involvement. Moreover the titres of these antibodies fluctuate with disease activity and therefore serial testing is a useful monitoring tool. Typically, a disease flare is accompanied by a rising titre of dsDNA antibodies and erythrocyte sedimentation rate (ESR), and falling complement and lymphocyte count. The C-reactive protein (CRP), unlike the ESR, does not usually rise with disease activity unless there is arthritis or serositis, and a raised CRP in a patient with SLE must always make you consider infection.

## Treatment

SLE is a relapsing and remitting disease, and treatment aims are threefold: managing acute periods of potentially life-threatening ill health, minimizing the risk of flares during periods of relative stability, and controlling the less life-threatening, but often incapacitating day to day symptoms. Our limited understanding of the precise pathogenesis of SLE means that the majority of treatments are still broadly immunosuppressive in action, and hence carry a significant risk of adverse effects.

At the milder end of the spectrum, hydroxychloroquine is commonly used. This is effective for skin disease, joint pain and fatigue. Non-steroidal anti-inflammatory drugs are also useful for arthralgia and arthritis, though more aggressive treatment with methotrexate may be required. Low dose oral steroids or intramuscular injections of depot steroid preparations are sometimes used for mild disease, but immunosuppressive therapies and high dose steroids are generally reserved for major organ involvement.

Lupus nephritis remains the complication which carries with it the biggest risk of death or long-term morbidity. Treatment of renal disease (Cochrane review [17]) was standardized by the National Institute of Health guidelines [18] published in 1992. Combining high dose corticosteroids with cyclophosphamide was the gold standard in the management of proliferative lupus nephritis for many years. Although efficacious, this regimen is limited by significant toxicity. Both agents are immunosuppressive. In addition, corticosteroids are associated with a whole host of adverse effects including osteoporosis and weight gain, and cyclophosphamide can cause haemorrhagic cystitis and infertility. More recently, the classic regimen of monthly boluses of 1g cyclophosphamide for 6 months, followed by once every three months for the next 2 years, has been modified by some groups, who instead advocate the use of "low-dose" cyclophosphamide (6 fortnightly pulses of 500 mg). The so-called Euro-lupus trial, published in 2002, showed that the use of this lower dose regimen has better outcomes in terms of infertility risk, with no deleterious impact on renal disease [19]. Following remission induction, azathioprine is commonly used for maintenance therapy. Mycophenolate mofetil [20] has been added to the repertoire of drugs used for the treatment of lupus nephritis. This is now used commonly as maintenance therapy following cyclophosphamide, and its use in the induction phase has been adopted in some centres.

Similarly, immunosuppressive treatments, such as cyclophosphamide and azathioprine, are also used for central nervous system involvement and rarely, serositis and haematological disease. Furthermore, persistent autoimmune thrombocytopenia sometimes requires immunoglobulin.

In an attempt to improve management, biological therapies are being developed, which target specific cells or molecules within the abnormally functioning immune system. For example, the depletion of B cells using rituximab, an anti-CD20 monoclonal antibody previously used in the treatment of B cell lymphomas, is now being used in patients with severe disease which has not responded to conventional treatments [21].

## Prognosis

Despite significant advances in treatment over the last decade, SLE still caries a significant risk of mortality and long term morbidity. A European study of 1000 patients with SLE, demonstrated a 10 year survival probability of 92% overall, reduced to 88% in those who presented with nephropathy [3]. Mean age at death was 44, but varied widely from 18–81 years.

Cause of death varies with disease duration. In one cohort [22]), renal lupus accounted for the biggest number of deaths in those with less than 5 years of disease, whereas vascular disease was the most important factor in the group who died later in the disease course.

As mentioned previously, we are becoming increasingly aware of the impact that premature atherosclerosis is having on the long term prognosis of lupus patients who survive the early years of illness. As we develop better immune targeted therapies, optimizing the management of these longer term complications will become increasingly important.
